# A flavour perspective of Tiepishihu (*Dendrobium officinale*) – an emerging food ingredient from popular traditional Chinese medicinal plants: a review

**DOI:** 10.1111/ijfs.16608

**Published:** 2023-08-16

**Authors:** Aidan Kirkwood, Ian Fisk, Charfedinne Ayed, Yingjian Xu, Ni Yang

**Affiliations:** ^1^ Division of Food, Nutrition and Dietetics University of Nottingham, Sutton Bonington Campus Loughborough LE12 5RD UK; ^2^ The University of Adelaide North Terrace Adelaide South Australia Australia; ^3^ Golden Keys High‐Tech Materials Co., Ltd First and Second Floor, Building No. 3, Guizhou ChanTou Science and Tech Industrial Park, Hulei Road, Huchao Town Guian new Area Guizhou Province China; ^4^ Department of Chemistry University of Warwick Coventry CV4 7AL UK

**Keywords:** Aroma, environment, orchids, processing, taste

## Abstract

Many Dendrobium orchid stems are used in Traditional Chinese Medicine (TCM). The most popular and premium species is *Dendrobium officinale,* and its stem in TCM is called Tiepishihu. Tiepishihu has a sweet flavour and is an ingredient in Chinese tea and desserts. There is no comprehensive understanding of its flavour compounds. It is, therefore, essential to understand compounds responsible for its flavour, and how they are formed. This review assesses twelve diverse studies in Tiepishihu flavour (2013–2022). Thirty aroma compounds were compared – furfural and nonanal were identified as common compounds. Four of seven essential amino acids were taste‐active, with lysine being the most potent. Pre‐harvest factors such as environment impact specific aroma compounds. Post‐harvest processing methods, including drying and grinding, can control Tiepishihu's flavour. Methodological consistency is a challenge, but controlling Tiepishihu's flavour could increase its commercial value as a food ingredient.

## Graphical abstract

Word cloud depicting frequently used terms in the current review.

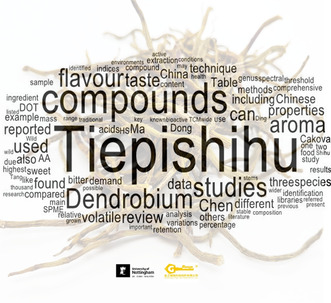

**Highlights**

- Important flavour compounds in Tiepishihu, an emerging food ingredient, are reviewed.
- At least thirty diverse volatiles compounds contribute to Tiepishihu's aroma.
- Furfural and nonanal were commonly reported aroma compounds in Tiepishihu.
- Lysine was proposed to be the most potent taste‐active amino acid in Tiepishihu.
- Pre‐harvest and post‐harvest process conditions are critical to control Tiepishihu flavour.

## Introduction

**Figure 1:**
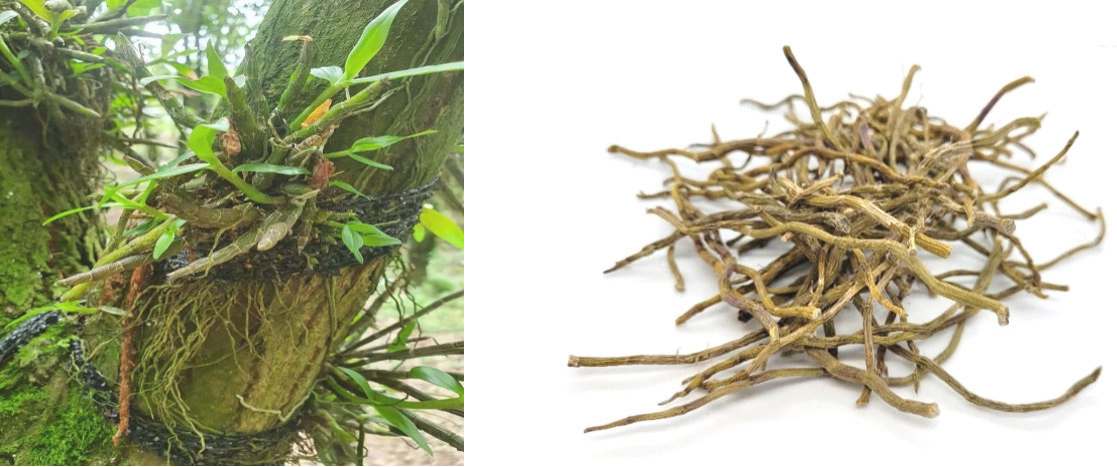
Author's photographs of *Dendrobium officinale* in plant and dried Tiepishihu form.

Dendrobium is a genus of epiphytic orchid belonging to the Orchidaceae family that grows in East Asia. This genus consists of over a thousand species of orchids, with more than seventy species found exclusively in China (Cheng *et al*., 2019). Dendrobium have fragrant and waxy flowers with lanceolate leaves, and its stems are thick and articulated. Over half of the Dendrobium species are used in Traditional Chinese Medicine (TCM) and are referred to as ‘Shihu’ (Cakova *et al*., 2017; Chinese Pharmacopoeia, 2022). Stems from one species in particular, *Dendrobium officinale*, referred to as ‘Tiepishihu’ (Fig. 1) is well respected as one of the most expensive herbs from the Dendrobium genus (Ye *et al*., 2017). The cost for the highest quality of Tiepishihu in 2002 (Ding *et al*., 2002) was 3000 USD per kg. The use of Tiepishihu dates back to the oldest recorded Chinese herbal collection, Shennong Ben Cao Jing (25–220 AD), and has been traded and consumed for over a thousand years (Teoh, 2016). Tiepishihu is also a component of Chinese tea and modern cuisine, known for its “sweet” flavour (Zhu *et al*., 2018).

In TCM, Tiepishihu is used to treat minor ailments like indigestion, dehydration, and fever (Tang *et al*., 2017). At present, much of the research has centered around the chemical profiling of Tiepishihu and the identification of possible bioactive compounds that explain the herb's traditional medicinal effects. Bioactive compounds (polysaccharides, phenols, and alkaloids) have been identified in Tiepishihu by many studies (including Cakova *et al*., 2017; Huang *et al*., 2015), including an *O*‐acetylglucomannan polysaccharide (dendronan) which has been identified in Tiepishihu and is believed to be the key bioactive compound.

Commercially Tiepishihu is an ingredient in nutraceutical beverages and health foods in China (Meng *et al*., 2019). Tiepishihu is developing a wider range of consumer applications, including use in stir‐fries and sauces, teas, juices, wine, and desserts (Cakova *et al*., 2017). The presence of glucomannans and pectin could give Tiepishihu water absorbing, thickening, and emulsifying properties when used as a food ingredient (Devaraj *et al*., 2019).

Wild Dendrobium is listed by the Convention on International Trade in Endangered Species of Wild Fauna and Flora as an ‘endangered’ plant but high demand in China has encouraged research centres to promote the restoration and conservation of Dendrobium (Tang *et al*., 2017). Dendrobium can be successfully grown in a wide range of environments and under climate‐controlled greenhouses is improving. Increased demand has meant that China has been sourcing Dendrobium from nearby countries like Laos, Myanmar, Nepal and Vietnam, which is also helping to restore wild Chinese Dendrobium (Hinsley *et al*., 2018). Methods such as backpropagation neural networks and programmable logic controllers (Ding *et al*., 2018) can be used to maintain the wild conditions of Dendrobium. Greenhouse cultivation may be therefore key to ensuring a stable supply to sustain the growing global demand of Tiepishihu.

For wider consumers to accept Tiepishihu as a food ingredient, it is important to have an appealing flavour that is both consistent and stable. There have been previous studies that have shown the volatile composition of Tiepishihu, but little has been done in terms of flavour characterisation.

This review will focus on Tiepishihu because it is in popular demand in China, it is seen as a more premium Dendrobium product, and it allows direct comparison between studies of one species. Therefore, a comprehensive understanding of its flavour properties is useful to evaluate how future commercial growth environments reflect the flavour properties of traditionally grown Tiepishihu. To achieve this, this review examined all the available scientific literature (2013–2022) related to flavour‐active compounds found in Tiepishihu and proposed comprehensive strategies for comprehending, controlling, and optimising flavour through various means, including genetic and epigenetic modifications, environmental adjustments, traditional agricultural practices, and simple post‐harvest processing techniques.

## Flavour profile of Tiepishihu

This review covers two main groups of flavour compounds reported in Tiepishihu: volatile compounds that can be perceived by olfactory receptors in the nose, and non‐volatile compounds that are tastants predominantly perceived by gustatory receptors on the tongue.

### Volatile aroma compounds in Tiepishihu

Identification of volatile compounds in Tiepishihu is of importance to flavour analysis. To date, more than 250 volatile compounds have been reported in Tiepishihu. Three studies on its volatile analysis and aroma compounds were included and compared in Table 1. Different numbers of aroma compounds from Tiepishihu were reported: from eleven (Ma *et al*., 2018) to over thirty (Chen *et al*., 2016; Dong *et al*., 2020). When all studies were compared, a total of thirty aroma compounds were listed (Table 1), which could be classified as aldehydes (8), alcohols (9), acids (4), ketones (4), and others (5). Main compounds (in bold) were chosen as the top three relative percentage content for each study. For example, furfural (*bready*), eucalyptol (*herbal*), and hexanal (*green*) are the main compounds from Chen *et al*. (2016). Comparing all three studies, only two aroma compounds were found to be in common: furfural, nonanal (*aldehydic*). The remaining twenty eight compounds varied significantly between reports, apart from the different pre‐harvest conditions among samples, the main variations between the studies could be due to different methods in sample preparation and extraction, analytical instrument parameters, and methods for compound identification and authentication. This review summarised three major sources of variations when comparing these studies:

First, different sample preparations might have led to different aroma compounds being reported. The study by Chen *et al*. (2016) defined the samples as freshly picked stems, whilst this information was not provided by others. Indication of sample freshness is important as changes to aroma composition might occur throughout shelf‐life and exposure to light (Sigrist *et al*., 2002). There was also variation in replicate numbers Chen *et al*. (2016) and Ma *et al*. (2018) used two to three replicates for Gas Chromatography–Mass Spectrometry (GC–MS) analysis, other studies did not report the number of replicates.
Second, each study used a different aroma extraction technique: headspace sampling (Chen *et al*., 2016), headspace‐solid‐phase microextraction (HS‐SPME) (Ma *et al*., 2018), and thermal desorption (Dong *et al*., 2020. Each technique has advantages and disadvantages. For example, HS‐SPME, as a quick and solvent‐free technique, can isolate volatiles and semi‐volatiles, but only semi‐quantitative data can be obtained. Whilst solvent extraction can provide more quantitative data with established solid methods, this technique can be more time‐consuming than the HS‐SPME technique (Hu *et al*., 2019).
Finally, differences in data analyses could lead to challenges in comparing results. For example, some of the three studies employed the use of (i) mass spectral libraries and retention indices (Ma *et al*., 2018) and (ii) mass spectral libraries, retention indices, and authentic standards (Chen *et al*., 2016), whilst another did not comment on the identification methods used (Dong *et al*., 2020). Lack of comprehensive assignment could lead to reporting incorrect aroma compounds. Whilst mass spectral libraries and retention indices can tentatively assign a compound's identity, authentic standards are normally required for full confirmation. There was also variation in the method of data presentation, for example, some authors expressed data as percentage relative content (Dong *et al*., 2020), and others expressed data as a percentage total peak area (Chen *et al*., 2016; Ma *et al*., 2018). To ensure maximum comparability across studies, the relative content (%) was used herein and compared in Tables 1 and 2.

**Table 1:** Summary of aroma compounds isolated from *Dendrobium officinale* stems from three published studies (1, 2, 3)

						Reference			
Functional group	No	Common name	CAS	MW	Odour reference^#^	1	2	3	Range (% content)
Aldehydes	1	Furfural	98‐01‐1	96	Bready	**X**		X	0.17–20.84
	2	Hexanal	66‐25‐1	100	Green	**X**			8.59
	3	Benzaldehyde	100‐52‐7	106	Fruity	X			1.28
	4	Phenylacetaldehyde	122‐78‐1	120	Green	X			6.50
	5	(E)‐2‐octenal	2548‐87‐0	126	Fatty		**X**		3.33
	6	Nonanal	124‐19‐6	142	Aldehydic	X	**X**		0.23–3.55
	7	p‐menth‐1‐en‐9‐al	29548‐14‐9	152	Spicy, herbal	X			0.34
	8	Decanal	112‐31‐2	156	Citrus		X		2.98
Alcohols	9	2,3‐butanediol	513‐85‐9	90	Creamy	X			0.41
	10	3‐ethylphenol	620‐17‐7	122	Musty	X			5.33
	11	Furaneol	3658‐77‐3	128	Caramellic			X	0.56
	12	1‐octanol	111‐87‐5	130	Waxy	X			2.93
	13	Linalool	78‐70‐6	154	Floral	X			9.46
	14	Eucalyptol	470‐82‐6	154	Herbal	**X**			10.79
	15	4‐terpineol	562‐74‐3	154	Spicy	X			1.02
	16	Cedrol	77‐53‐2	222	Woody			X	0.81
	17	Phytol	150‐86‐7	296	Floral				1.60
Acids	18	Acetic acid	64‐19‐7	60	Sour			X	5.23
	19	Octanoic acid	124‐07‐2	144	Fatty	X			2.81
	20	4‐methyloctanoic acid	54947‐74‐9	158	Fatty	X			0.79
	21	Myristic acid	544‐63‐8	228	Waxy		X		1.30
Ketones	22	Heptan‐2‐one	110‐43‐0	114	Cheesy	X			0.24
	23	Acetophenone	98‐86‐2	120	Floral	X			0.46
	24	Beta ionone	79‐77‐6	192	Floral		X		1.54
	25	Geranyl acetone	689‐67‐8	194	Rose		X		2.36
Others	26	Ethyl octanoate	106‐32‐1	172	Waxy	X			2.01
	27	2‐acetyl pyrrole	1072‐83‐9	106	Musty			X	0.50
	28	Naphthalene	91‐20‐3	128	Moth balls		**X**		4.27
	29	Terpinolene	586‐62‐9	136	Herbal	X			5.62
	30	Longifolene	475‐20‐7	204	Woody		X		0.41

Relative concentration was reported from published data: 1—Chen *et al*. (2016), 2—Ma *et al*. (2018) and 3—Dong *et al*. (2020). Characters and number in bold indicated main compounds (top three peak areas). Table excluded compounds that were a) not volatile at atmospheric pressure, i.e. above 300 molecular weight MW, and b) had no recognised aroma properties in literature.

^#^ Odour references from The Good Scents Company (2022).

**Table 2:** Aroma compounds isolated from purple (P) and green (G)‐coloured *D. officinale* stems from four provinces in China: Zhejiang (Z) at East, Fujian (F) at Southeast, Yunnan (Y) at Southwest and Jiangxi (J) at Southeast

Functional group	No	Common name	CAS	MW	Odour reference^1^	Relative content (%)^#^					
						Zhejiang (Z)		Fujian (F)		Yunnan (Y)	Jiangxi (J)
						Z‐P	Z‐G	F‐P	F‐G	Y‐P	J‐P
Aldehydes	1	Hexadecanal	629‐80‐1	240	Cardboard				0.20		
	2	Octadecanal	638‐66‐4	269	Oily	0.41	**0.83**	0.46	0.71	**0.82**	0.38
Alcohols	3	1‐hexadecanol	36653‐82‐4	242	Waxy	**0.63**	0.39		0.57	0.13	0.48
	4	Phytol	150‐86‐7	297	Floral	**2.26**	**1.17**	**3.05**	**3.05**	**0.95**	**3.23**
Acids	5	Myristic acid	544‐63‐8	228	Waxy	0.22	0.18	0.32	0.43	0.19	0.26
	6	Pentadecanoic acid	1002‐84‐2	242	Waxy	0.55	0.37	**1.18**	**1.15**	0.79	**0.68**
Ketones	7	Camphor	76‐22‐2	152	Camphoreous	0.17		0.22		0.20	0.19
	8	Hexahydrofarnesyl acetone	502‐69‐2	269	Floral					0.12	
Others	9	Ethyl palmitate	628‐97‐7	284	Waxy			0.23	0.29		
	10	Dihydroactinidolide	15356‐74‐8	180	Fruity					0.13	
	11	Delta‐tetradecalactone	2721‐22‐4	226	Waxy				0.17	0.16	

Characters and number in bold indicated main compounds (top two peak areas). Compounds were identified using NIST Mass Spectral Libraries and identified using Retention Indices in literature. Table excluded compounds that were a) not volatile at atmospheric pressure, *i.e*. above 300 molecular weight MW, and b) had no recognised aroma properties in literature.

^1^ Odour references from The Good Scents Company (2022).

^#^ Relative content (% of peak area) was reported in published data (Hu *et al*., 2020).

Overall, previous work has provided preliminary results on Tiepishihu's aroma profile. However, due to the mentioned variations between the studies, it is difficult to fully understand the flavour profile of Tiepishihu. There are no Tiepishihu (or even Shihu for that matter) studies to date that have determined odour activity, so the impact of aroma compounds cannot be considered. For odour activity, this could include using Gas Chromatography‑Olfactometry (Lester *et al*., 2021).

### Non‐volatile tastants in Tiepishihu

Based on the recently reported results in Tiepishihu, this section of the review focuses on amino acids, polyphenols, and sugars that could potentially contribute to the taste of Tiepishihu.

#### Amino acids and polyphenols

Amino acids (AA) are crucial for nutrition, health, but also for their diverse taste properties,  for example sweetness, albeit less potent than sugars. Essential AA must be obtained from food as the body cannot synthesise them. Currently, seven out of the nine essential AA have been reported in Tiepishihu (Yuan *et al*., 2019). The taste properties of these seven AA vary from flat to sweet and bitter (Table 3). This review has calculated dose‐over‐threshold factors in accordance with the method described by Scharbert & Hofmann (2005). Dose‐over‐threshold (DOT), included in Table 3, evaluates the taste contribution of separate taste compounds. When the ratio of dose over threshold factor is ≥1.0, the compound is ‘taste active’. The present review found that four amino acids (threonine, methionine, isoleucine and lysine had a DOT ≥1.0 for all stem ages. Tiepishihu contained threonine at the highest abundance from the literature, but threonine has a relatively low DOT value, when compared to lysine, which has the highest DOT. It may be speculated that the “bitter, complex, salty, sweet” taste of lysine contributes to the sweet taste of Tiepishihu.

**Table 3:** Summary of seven essential amino acids identified in stems of *Dendrobium officinale* (2–3 years old) with their taste descriptions, thresholds and dose‐over‐threshold (DOT) values for species

Amino acid	Taste descriptions^1^	Taste threshold (mg/g) in water^2^	Mean concentration (mg/g of dry weight)^3^ [DOT factors]^4^	
			2 years	3 years
Threonine	“Flat to sweet, possibly bitter, sour, or fatty”	3.06	6.16 [2.0]	3.54 [1.2]
Valine	“Flat to bitter; slightly sweet”	0.49	0.46 [0.9]	0.26 [0.5]
Methionine	“Flat to bitter; possibly complex and strangling”	0.56	0.6 [1.1]	0.6 [1.1]
Leucine	“Flat to bitter (virtually indistinguishable from L‐isoleucine)”	0.85	0.48 [0.6]	0.36 [0.4]
Isoleucine	“Flat to bitter”	0.97	1.94 [2.0]	1.3 [1.3]
Phenylalanine	“Bitter; possibly complex and strangling”	1.09	0.92 [0.8]	0.6 [0.6]
Lysine	“Bitter, complex, salty, sweet”	0.1	1.26 [12.6]	0.94 [9.4]

^1^ Taste descriptions of each amino acid's L‐enantiomer.

^2^ Taste thresholds in deionised water were obtained from Schiffman & Sennewald (1981).

^3^ Mean concentration values (mg/g of dry weight) were calculated from published data (Yuan *et al*., 2019).

^4^ DOT factor calculated by the division of amino acid concentration in sample (mg/g) by taste threshold (mg/g).

Polyphenols are also of interest due to their bitter, astringent, and antioxidant properties (Cakova *et al*., 2017). The only possible polyphenol tastant to be found in Tiepishihu is quercetin. Quercetin is found in a wide variety of fruit and vegetable produce and is known for its bitter flavour and cardioprotective effects (Patel *et al*., 2018). An optimised extraction by Zhu *et al*. (2019) quantified quercetin (2.506–2.594 μg/g) in Tiepishihu. The taste threshold for quercetin (in 5% ethanol), presented in different units, is 4.53 μg/mL (Dresel *et al*., 2015). As the DOT factor is ≤1.0, quercetin is not likely to be taste‐active in the Tiepishihu extract by Zhu *et al*. (2019).

#### Sugars

A range of free sugars have been detected in Tiepishihu, and it would be valid to speculate that sugars could contribute to the ‘sweet’ nature of the extract. A study by Jin *et al*. (2016) analysed derivatised methanolic extracts of Tiepishihu using GC–MS. Among the twenty‐six sugars and glycosides reported, it is worth noting that sucrose, glucose, galactose, and fructose were identified. The presence of these well‐established sweet‐tasting monosaccharide and disaccharide sugars is likely to contribute to the sweet nature previously used to describe Tiepishihu, but future work into quantitative analysis could indicate whether these sugars are present at taste‐active concentrations.

#### Other possible tastants

There may be more tastants in Tiepishihu, for example, alkaloids (dendrobine, Cheng *et al*., 2019), coumarins (Jin *et al*., 2016), and aromatic acids (ferulic acid, Ye *et al*., 2017). At similar concentrations, which contribute taste/flavour to other plant systems. However, the lack of quantitative data and taste threshold references makes it difficult to evaluate their contribution to Tiepishihu's taste.

Overall, many non‐volatile compounds can possess gustatory properties, and from the available comprehensive data, essential AA may be contributing to the taste of Tiepishihu. Only essential AA have been measured and reported, however, non‐essential AA also have taste properties. Sugars, amino acids, polyphenols, sugars, alkaloids, coumarins, and aromatic acids may contribute to taste, and therefore, future work should involve quantification and taste threshold studies.

## Factors affecting Tiepishihu's flavour profile

### Pre‐harvest

The major pre‐harvesting factors that may contribute to the flavour of Tiepishihu covered in this review are intrinsic factors (plant age; stem colour) and extrinsic factors (geography, altitude). The results from different studies (Yuan *et al*., 2019; Hu *et al*., 2020) are assessed, and their impact on Tiepishihu's flavour profile is summarised.

#### Intrinsic factors

##### Plant age

Dendrobium stems lignify as they age, providing structural support and hydrophobicity for the plant to grow tall and absorb nutrients (RHS Plants, 2022; Zhao, 2016). Yuan *et al*. (2019) quantified seven essential AA in the stems of 2‐ and 3‐year‐old *D. officinale* and the results showed a lower concentration (1.2%–50.0% decrease) of AA between 2‐year‐old and 3‐year‐old stems (Table 3). Threonine and valine content almost halved between two and three years. There was a significant decrease of five out of seven AA (threonine, valine, leucine, isoleucine, phenylalanine) after three years (*P* < 0.05), which was also the case for polysaccharide content (Yuan *et al*., 2019). Lignification could explain this reduction in phenylalanine as lignin biosynthesis starts with deamination of phenylalanine (Zhao, 2016). In terms of AA aroma generation, the stems of Dendrobium grown for different lengths of time may possess different aroma properties, so further study could follow from the work by Yuan *et al*. (2019).

##### Stem colour

Stem colour may have an impact on the aroma profile of Tiepishihu. Apart from the volatile analyses of red and green Tiepishihu stems by Hu *et al*. (2020), no other sources mention stem colours. *Dendrobium candidum*, on the other hand, is better known for its green and red stems—the red ones are said to be more expensive and a predictor of good quality (Jia *et al*., 2021). Anthocyanins, specifically, cyanin and cyanidin, have been found to give the red pigmentation in *D. candidum*, which is mediated through the transcription factor DcTT8 (Jia *et al*., 2021). The extrinsic factors that affect its expression of anthocyanins has have not yet been devised, but the drought has been shown to cause transcription factor‐induced anthocyanin expression in apples (An *et al*., 2020). Extrinsic factors are not the only reason for red pigmented stems: in some plants, like Hedera helix, red stems are characteristic of juvenile plants (Hackett, 2002). In *D. officinale*, interestingly, camphor (minty) was only detected in purple stems in all four locations but not green ones (3). The mechanism between stem colour and camphor production cannot be proposed, but it could be that camphor (a pest deterrent) is generated in response to stress. Green stems from Fujian had a unique aroma compound—hexadecanal (cardboard), and dihydroactinidolide (floral, rose) only detected in purple stem from Yunnan. There is no statistical difference in the relative content of volatiles in different coloured stems from the same provinces. It is not known whether the same anthocyanins in *D. candidum* leads to purple stems of Tiepishihu, and therefore, further work is required to determine: (i) the pigmented compounds in Tiepishihu, (ii) factors contributing to pigmentation (such as drought), and (iii) whether colouration causes a difference in flavour quality.

#### Extrinsic factors

##### Geography

Dendrobium is mainly distributed in the sub‐tropical regions in China, including Guizhou, Yunnan, and Guangxi (Xiong *et al*., 2022). The impact of region on the volatile composition of Tiepishihu has been reported by Hu *et al*. (2020). The study profiled the composition of dried stems from four provinces in China using qualitative GC–MS and reported 101 volatiles from the six types of samples analysed. Further analysis into the supplementary of Hu *et al*. (2020) showed the results of eleven aroma compounds in total (Table 2). Six out of eleven aroma compounds are common between different locations, and four compounds appeared in all samples: octadecanal (*oily*), phytol (*floral*), myristic acid (*waxy*), and pentadecanoic acid (*waxy*). Among the compounds in bold with higher relative content for each sample, phytol had the highest levels (0.95%–3.23%). In comparing four locations, Tiepishihu from Yunnan had a larger number of aroma compounds detected with two unique fruity, *floral* aroma compounds (hexahydrofarnesyl acetone and dihydroactinidolide) and ethyl palmitate (*waxy*) only appeared in samples from Fujian. Different locations in China offer various environmental stresses for growing Dendrobium due to altitude, temperature, soil pH and composition, UV exposure, and climate. Volatile composition changes by geography offer consumers more choices and preferences.

##### Altitude

Altitude affects herb growth and extract quality via temperature, humidity, wind, water, and sunlight differences. Dendrobium is typically grown at an altitude of 1600 m (Guo *et al*., 2020) above sea level in parts of China (tropics and subtropics). In a study (Do Carmo *et al*., 2020), a significant (*P* < 0.05) positive relationship was found between the altitudes for growing Arabica coffee (*Coffea arabica*) and resulting aroma with higher sensory scores for aroma, flavour, and acidity in coffee growth at a higher altitude (1050 vs. 850 m). It has been shown previously that environmental conditions at higher altitudes can modulate the transpiration and photosynthesis rates of coffee plants, which leads to a more delicate coffee aroma (Hameed *et al*., 2020). Therefore, the aroma qualities in different plant extracts can be affected either positively or negatively by growth altitude. These differences are plant‐specific, and there is a gap in the knowledge of the effect of altitude on the quantitative aroma composition and quality of Tiepishihu products.

### Post‐harvest process

**Figure 2:**
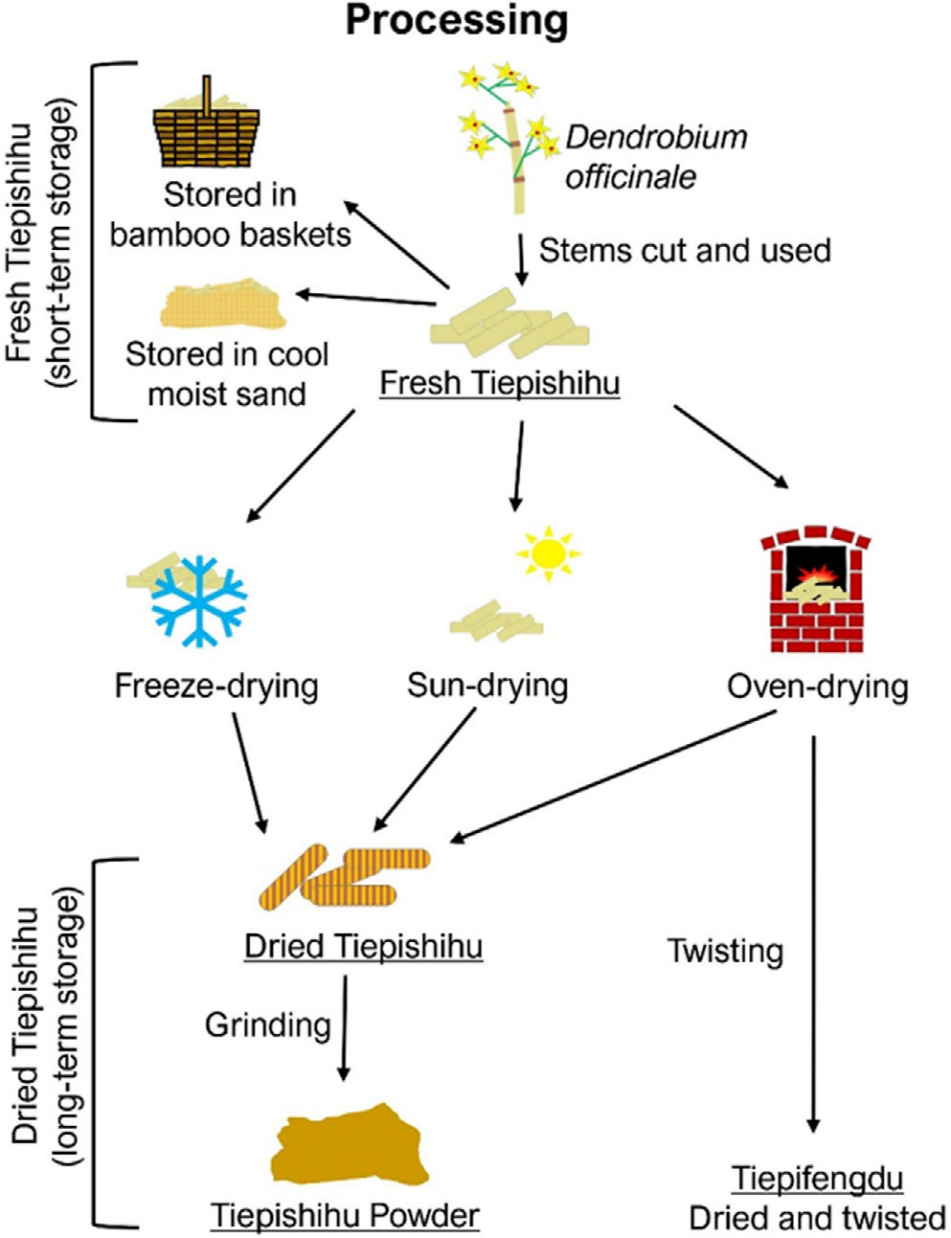
Author's representation and classification of known post‐harvesting Shihu techniques based on Teoh (2016) and Cao *et al*. (2020). Products are underlined.

Traditional post‐harvest processes involved in Tiepishihu production can be split into five stages: cutting, fresh storage, drying, twisting, and grinding (Fig. 2). Dendrobium stems are harvested and separated, then stored short or long‐term. Short‐term storage is in bamboo baskets or cool sand. For long‐term storage, stems are rinsed and freeze‐, sun‐, or oven‐dried. Dried Tiepishihu comes in various forms, as shown in Fig. 2 (Teoh, 2016). For long‐term storage, stems are rinsed with water and then freeze‐, sun‐, or oven‐dried, for preservation.   The dried and twisted form for Tiepishihu is named Tiepifengdou (Cao *et al*., 2020). Tiepishihu flavour may be controlled by these post‐harvesting factors, such as different drying and grinding conditions.

#### Drying

No studies have evaluated how each post‐harvest step can affect the flavour profiles of Tiepishihu. Drying can play a particularly important role, because it reduces water content, limits microbial growth, and inhibits enzymatic reactions, which in turn leads to longer shelf life (Orphanides *et al*., 2015). Drying methods can influence the aroma compositions of herbal extracts, such as basil and coriander (Ghasemi Ghasemi Pirbalouti *et al*., 2013; Ghasemi Pirbalouti *et al*., 2017), however, the optimal drying parameters are not clear with Tiepishihu. Some studies (Chen *et al*., 2016; Dong *et al*., 2020) indicated that Tiepishihu was freeze‐dried and oven‐dried at 40 °C, respectively. Freeze‐drying seems to reduce oxygenated terpenes and sesquiterpenes in spearmint (*Mentha spicata*) leaves, due to the expansion of surface layer cells. Qualitative data of Tiepishihu makes it difficult to know these have been lost (Consuelo Díaz‐Maroto *et al*., 2003). The presence of furfural in freeze‐dried Tiepishihu stem (Chen *et al*., 2016) is interesting since furfural formation is typically associated with thermal processing – through dehydration of pentose sugars (Spinnler, 2011). A higher drying temperature of 65 °C (for 2 days) in the study by Ma *et al*. (2018) may explain the fewer compounds identified compared to others, potentially through volatilisation.

One study described a ‘standard method’ for Tiepishihu drying at 50 °C for 24 h (Cao *et al*., 2020), one for as long as 15 days (at 50 °C) (Hu *et al*., 2020) and another for 7 days (at 30 °C) (Pan *et al*., 2015), but these studies did not apply volatile analyses, and so the impact on aroma properties is unknown. The present review, therefore, recommend standardising drying protocols.

#### Grinding

Dried Tiepishihu is also processed into a ground form, which is often sold on the Chinese market as a tea ingredient. Superfine grinding of Tiepishihu increases the solubilities of its constituents (polysaccharides, proteins, and antioxidants) (Meng *et al*., 2019). Importantly, superfine Tiepishihu powder (optimal particle size of 30 μm) requires less time to dissolve compared to higher particle sizes (Meng *et al*., 2019). This can have a positive impact on the consumer, as shortening preparation time is convenient. No study has shown the impact of Tiepishihu particle size on flavour extraction, however, with horseradish (*Armoracia rusticana*) powders, ball milling for longer times (15–60 min) increases isothiocyanate content (Wang *et al*., 2020). From a mechanistic perspective, grinding for longer results in lower particle size and increased surface area. This can lead to reactions (*i.e*., enzymatic hydrolysis), which can alter the composition and proportion of volatile compounds. They found that refrigerated ball‐milling (6 °C) could preserve volatile aroma compounds (Wang *et al*., 2020).

## Conclusion

Tiepishihu, the stems of *Dendrobium officinale*, is an important growing premium Traditional Chinese Medicine with the potential to be a food ingredient of international commercial importance. This review summarises recent literature on the flavour composition of Tiepishihu.

From a flavour perspective, recent studies on volatile and non‐volatile compounds present in Tiepishihu were compared. Thirty volatiles were identified from previous studies such as furfural and nonanal. This review article identified that AA are the only taste‐active components in Tiepishihu (for example, lysine). Sugars like glucose might be contributing to the sweet taste of Tiepishihu, but in these studies, many lacked quantification so its true taste impact might be overlooked.

Factors that impact flavour composition include pre‐harvesting factors (plant age, geography, and altitude regions for Dendrobium growth) and post‐harvesting factors (*i.e*., drying and grinding conditions). Plant age affects the amino acid content in Dendrobium stems, with older stems showing lower concentrations of amino acids. Stem colour may also impact the aroma profile, with certain compounds detected only in specific‐coloured stems. Different drying methods and conditions can alter the aroma compositions, but the optimal parameters for Tiepishihu are still unclear. Reliable and consistent methods were recommended for future investigation of flavour composition so that the impact of cultivation and processing methods on the flavour of Tiepishihu products can be better understood.

Due to gaps identified in the current studies, this review proposed improved methodologies to quantify and identify the key odour‐active compounds (through GC‐Olfactometry) in future studies. More studies on advanced and novel food processing techniques will help create innovative Tiepishihu products of high quality with desirable flavours, enabling their wider food applications in the global market.

## Author contributions

**Aidan Kirkwood:** Conceptualization (equal); investigation (lead); writing – original draft (lead). **Ian Fisk:** Conceptualization (equal); supervision (equal); writing – review and editing (equal). **Charfedinne Ayed:** Supervision (equal); writing – review and editing (equal). **Yingjian Xu:** Conceptualization (equal); supervision (equal); writing – review and editing (equal). **Ni Yang:** Conceptualization (equal); funding acquisition (lead); supervision (lead); writing – review and editing (equal).

## Funding statement

This work was supported by GoldenKeys High‐Tech Materials Co., Ltd. (贵州金之键高科技材料股份有限公司, 91520900MA6DL1ER7N, 黔石科合 [2019007]), and the Biotechnology and Biological Sciences Research Council (BBSRC) [grant number BB/V017284/1].

## Conflict of interest

The authors declare that they have no competing interests.

## Ethical approval

Ethical approval was not required for this research.

### Peer review

The peer review history for this article is available at https://www.webofscience.com/api/gateway/wos/peer‐review/10.1111/ijfs.16608.

## Data Availability

The data that support the findings of this study are available on request from the corresponding author.
